# Stk38 Modulates Rbm24 Protein Stability to Regulate Sarcomere Assembly in Cardiomyocytes

**DOI:** 10.1038/srep44870

**Published:** 2017-03-21

**Authors:** Jing Liu, Xu Kong, Lee Yew Mun, Zhang Meng Kai, Guo Li Yan, Yu Lin, Teck Kwang Lim, Qingsong Lin, Xiu Qin Xu

**Affiliations:** 1 The Institute of Stem Cell and Regenerative Medicine, Medical College, Xiamen University, 361100, P.R. China; 2 Department of Biological Sciences, National University of Singapore, 117543, Singapore; 3 Fujian Key Laboratory of Organ and Tissue Regeneration, Medical College, Xiamen University, 361100, P.R. China

## Abstract

RNA-binding protein Rbm24 is a key regulator of heart development and required for sarcomere assembly and heart contractility. Yet, its underlying mechanism remains unclear. Here, we link serine/threonine kinase 38 (Stk38) signaling to the regulation of Rbm24 by showing that Rbm24 phosphorylation and its function could be modulated by Stk38. Using co-immunoprecipitation coupled with mass spectrometry technique, we identified Stk38 as an endogenous binding partner of Rbm24. Stk38 knockdown resulted in decreased Rbm24 protein level in cardiomyocytes. Further studies using Stk38 kinase inhibitor or activator showed that Rbm24 protein stability was regulated in a kinase activity-dependent manner. Deficiency of Stk38 caused reduction of sarcomere proteins and disarrangement of sarcomere, suggesting that Stk38 is essential for Rbm24 to regulate sarcomere assembly. Our results revealed that Stk38 kinase catalyzes the phosphorylation of Rbm24 during sarcomerogensis and this orchestrates accurate sarcomere alignment. This furthers our understanding of the regulatory mechanism of cardiac sarcomere assembly in both physiologic and pathologic contexts, and uncovers a potential novel pathway to cardiomyopathy through modulating the Stk38/Rbm24 protein activity.

RNA-binding proteins (RBPs) are known to be involved in every step of RNA biology, including transcription, editing, splicing, transport and localization, stability, and translation^1^. RBPs play important roles in the regulation of gene expression during development and adulthood. Eukaryotic cells produce a large number of RBPs, each of which has unique RNA-binding activity and protein-protein interaction characteristics^2^. Growing interest in the functional repertoire of RBPs has emerged as their post-transcriptional regulatory mechanism has become more broadly appreciated. Tissue-specific RBPs have profound implications for cellular physiology, affecting RNA processes from pre-mRNA splicing to protein translation. Recent emerging evidences revealed that RBPs are involved in a broad spectrum of human diseases^3^. For example, Rbm20 was recently found to play a key role in the post-transcriptional regulation of cardiac function and was linked to pathogenesis of human cardiomyopathy and heart failure^4^,^5^.

Rbm24 (RNA Binding Motif Protein 24) is an RNA-binding protein. We previously identified it as a cardiac enriched gene product during human embryonic stem cell (ESC) cardiogenesis and subsequently characterized its role in heart development in a zebrafish model^6^,^7^. It is tissue-specifically expressed in the heart and muscle^7^,^8^. Most recently, we reported that Rbm24 played an important role in regulating ESC cardiac differentiation by a splicing-mediated regulatory mechanism^9^. Yang *et al*. reported that targeted inactivation of Rbm24 resulted in severe sarcomere disarray in mouse heart and led to embryonic lethality^10^. Furthermore, the authors found that Rbm24 functions as a major regulator in governing a large number of muscle-specific splicing events to regulate cardiac development^10^. Others demonstrated Rbm24 regulates p63^3^, p21^11^ and myogenin^11^ expression via mRNA stability^12^,^13^. These studies indicate Rbm24 plays important roles in transcription and post-transcriptional processing of RNA molecules during development. Nevertheless, the regulation of Rbm24 or its context signaling remains unknown.

Using co-immunoprecipitation coupled with mass spectrometry technique, we identified Stk38/NDR1 (Serine-threonine kinase 38/nuclear Dbf2-related protein kinase 1) as an endogenous binding partner of Rbm24. Stk38 belongs to a highly conserved family of kinases. From genetic and biochemical analysis, it has been shown that Stk38 kinase is activated by the phosphorylation of the hydrophobic motif, and its activated form in turn phosphorylates and activates downstream proteins^14^. Functionally, Stk38 kinases have been reported to regulate centrosome duplication^15^,^16^,^17^, apoptosis^18^,^19^, proliferation^20^, and chromosome alignment^21^. There were studies that demonstrated Stk38′s interaction with tumor suppressor Yap1 in Hippo pathway^22^. These highlight the important and diverse roles of Stk38 involved in controlling different physiological functions. However, Stk38′s function and pathway-related activity in the heart have yet to be reported.

Here, we report that Stk38 signaling links to the regulation of Rbm24 proteins in cardiomyocytes’ sarcomere assembly. Knockdown of Stk38 decreased the protein level of Rbm24, leading to the disruption of the highly-ordered structure of the cardiomyocyte cytoskeleton, and resulting in its irregular arrangement. Our studies have also defined a previously unknown biological role of Stk38 in modulating an RBP’s activity through affecting protein stability in a kinase activity-dependent manner, and document for the first time the ser/thr phosphorylation of Rbm24 by Stk38 kinase in the context of sarcomere assembly, thereby revealing a novel and unique regulatory mechanism of the Stk38-Rbm24 pathway in the heart.

## Results

### Identification of Rbm24-Stk38 interaction

**Figure 1:**
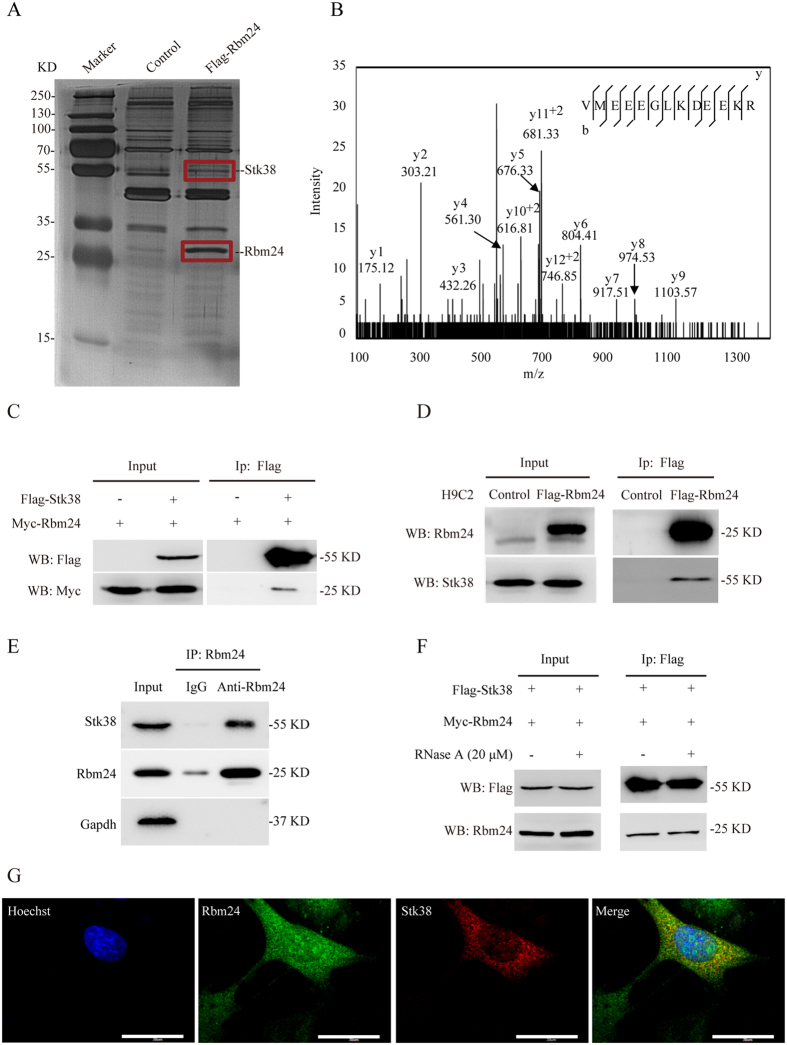
Stk38 directly interacts with Rbm24. (**A**) Pulldown assays with anti-Flag antibody-conjugated agarose beads were performed on lysates from H9C2-control and H9C2-Flag-Rbm24 cells. Bound proteins were eluted with Flag peptide and analyzed by silver staining. A unique protein band specific to the lysate of H9C2-Flag-Rbm24 cells was excised and identified with mass spectrometry to be the protein Stk38. The positions of Rbm24 and Stk38 on the gel were indicated to the right of the gel. (**B**) Representative MS/MS spectrum of a tryptic peptide (VMEEEGLKDEEKR) derived from Stk38. The assigned y ion peaks are indicated on the spectrum with respective m/z values. The b and y ions detected are indicated on the sequence. (**C**) Lysates from HEK293 cells transfected with Flag-Stk38 and Myc-Rbm24 were immunoprecipitated with anti-Flag antibody followed by western blot analysis. Immunoprecipitation with anti-Flag antibody showed that Rbm24 was pulled down along with Flag-Stk38, indicating their direct interaction. (**D**) Anti-Flag pulldown assays were performed from H9C2-control and H9C2-Flag-Rbm24 cells and analyzed by western blot using anti-Stk38 antibody. Immunoprecipitation with anti-Flag antibody showed that endogenously expressed Stk38 was pulled down along with Flag-Rbm24, further validating direct interaction between Rbm24 and Stk38.(**E**) Anti-Rbm24 pulldown assays were performed from HL-1 cells and analyzed by western blot using anti-Stk38 antibody. Immunoprecipitation with anti-Rbm24 antibody showed that endogenously expressed Stk38 was pulled down along with Rbm24. (**F**) Lysates from HEK293 cells transfected with Flag-Stk38 and Myc-Rbm24 were treated with RNase A, then immunoprecipitated with anti-Flag antibody before western blot analysis. Treatment with RNase A did not affect the interaction of Rbm24 with Stk38, indicating that their interaction is RNA-independent. (**G**) H9C2 was fixed and stained using anti-Stk38 (red fluorescence) and anti-Rbm24 (green fluorescence) antibody. Presence of overlapping fluorescence signal (yellow fluorescence) further indicated direct interaction of Rbm24 and Stk38 both in the cytoplasm and nucleus. Bar = 12 μm. The images of (**A,C,D,E,F**) shown are cropped. The full-length gel/blots or original images are shown in Fig. S1.

Previous studies showed that Rbm24 plays an important role in the regulation of cardiac development^6^,^7^,^10^. However, the molecular pathways employed by Rbm24 to exert its diverse cellular effects remain largely uncharacterized. To obtain more insight into Rbm24′s signaling complex and related functions, we established a H9C2 rat cardiomyoblast cell line that stably expresses a Flag-tagged version of human Rbm24 (H9C2-Flag-Rbm24) to pulldown proteins interacting with Rbm24, followed by silver staining and mass spectrometry analysis. H9C2 with integrated empty vector served as a control. As shown in Fig. 1A, a prominent 55-kDa band is present specifically in H9C2-Flag-Rbm24 lane following anti-Flag pulldown and silver staining. The band was excised and subjected to mass spectrometry analysis to identify Stk38 as the most significant hit, suggesting that Rbm24/Stk38 complex could be formed in the cells (Fig. 1B).

To test whether Rbm24 directly interacts with Stk38, we co-transfected Myc-Rbm24 and Flag-Stk38 into HEK293 cells. Co-immunoprecipitation experiments of Myc-Rbm24 with Flag-Stk38 showed that Rbm24 associated with Stk38 (Fig. 1C). The co-immunoprecipitation of H9C2-control and H9C2-Flag-Rbm24 with a Stk38-specific antibody also confirmed that Rbm24 could pulldown endogenous Stk38 in H9C2-Flag-Rbm24 cells (Fig. 1D). Moreover, using mouse HL-1 cell line, we demonstrated that endogenous Stk38 could be co-precipitated with Rbm24 when an anti-Rbm24 antibody was used for immunoprecipitation (Fig. 1E). Thus, the interaction between Rbm24 and Stk38 is validated under endogenous conditions.

Since Rbm24 is an RNA binding protein, we next examined if Rbm24/Stk38 interaction is RNA dependent using RNaseA treatment. As shown in Fig. 1F, despite with RNaseA treatment, Myc-Rbm24 could be pulled down by the Flag antibody from cells co-expressing Myc-Rbm24 and Flag-Stk38, suggesting a direct and RNA-independent interaction between Rbm24 and Stk38.

To further address the localization of the interaction, we sought to use H9C2 for direct immunofluorescence imaging. As shown in Fig. 1G, a significant proportion of Stk38 was found in the cytoplasm and nucleus, and the distribution of Stk38 and Rbm24 partially overlapped in the cytoplasm and nucleus regions (Fig. 1G).

### Both RRM and C-terminal domains are required for Rbm24 interaction with Stk38

**Figure 2:**
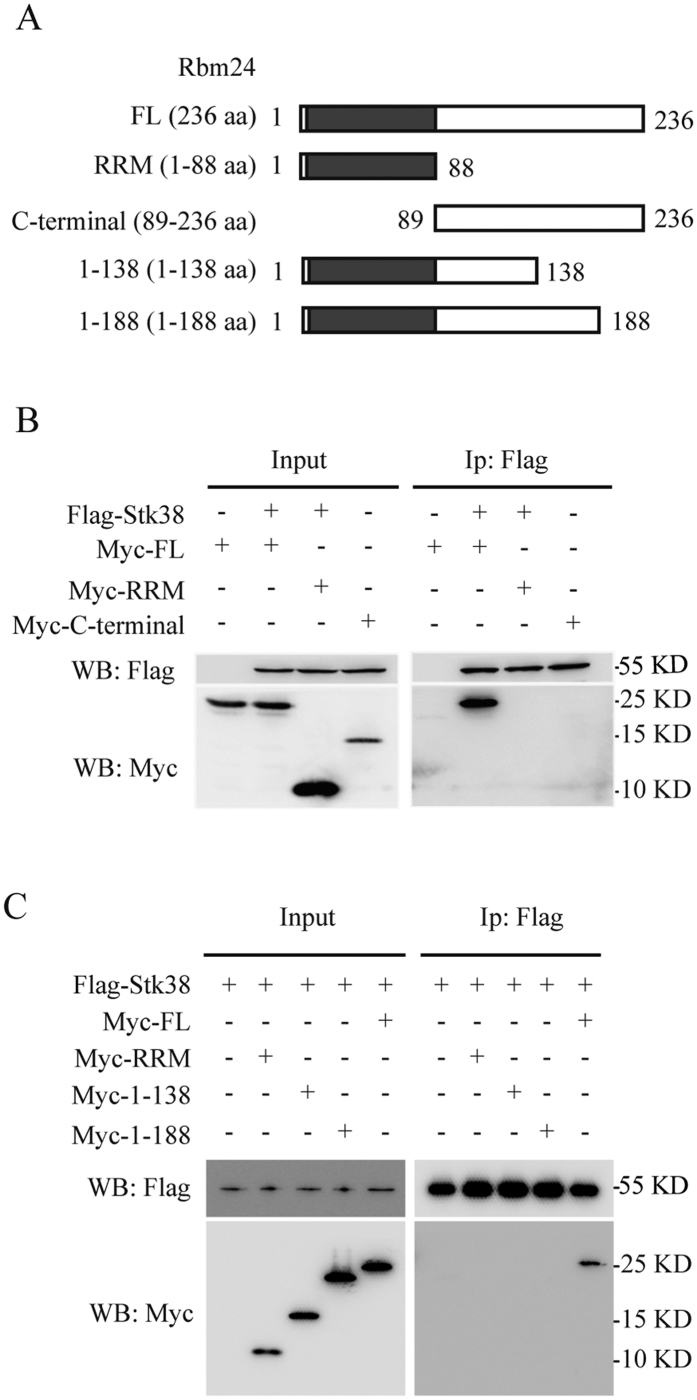
(**A**) Schematic representation of the structure of FL, RRM, C-terminal, 1-138 aa and 1-188 aa fragments of Rbm24. (**B**) Lysates from HEK293 cells transfected with Flag-Stk38 and Myc-FL/Myc-RRM/Myc-C-terminal of Rbm24 were immunoprecipitated with anti-Flag antibody followed by western blot. From the results, only Myc-FL was pulled down by Flag-Stk38, indicating that interaction between Rbm24 and Stk38 requires the full length Rbm24 to occur. (**C**) Lysates from HEK293 cells transfected with Flag-Stk38 and Myc-FL/Myc-RRM/Myc-1-138/Myc-1-188 of Rbm24 were immunoprecipitated with anti-Flag antibody followed by western blot. The images shown are cropped. The full-length blots or original images are shown in Figure S2.

A previous study showed that an N-terminal regulatory domain (NTR) of Stk38 provides a platform for protein-protein interaction^14^. For Rbm24, it contains a conserved RRM domain and a C-terminal domain^7^. However, the exact domain for Rbm24 protein-protein interaction is unknown. To assess which specific domain of Rbm24 is required for Rbm24/Stk38 interaction, three clones of HEK293 expressing different domains of Rbm24 (Myc-Rbm24, Myc-RRM or Myc-C-terminal; Fig. 2A) along with the full-length (FL) Stk38 were generated and used in pulldown assays. Figure 2B showed that Stk38 could pulldown Rbm24, but co-immunoprecipitation of Stk38 with Myc-RRM or Myc-C-terminal was not detected in these experimental settings. We investigated the interaction of Stk38 and Rbm24 in more detail using Rbm24 fragments 1–138 (amino acids 1 to 138) and 1–188 (amino acids 1 to 188) (Fig. 2A). Still, results showed that only full length of Rbm24 would pull down Stk38 (Fig. 2C), indicating that an individual domain of Rbm24 could not form a complex with Stk38.

### Knockdown of Stk38 affects Rbm24 protein stability and splicing activity

**Figure 3:**
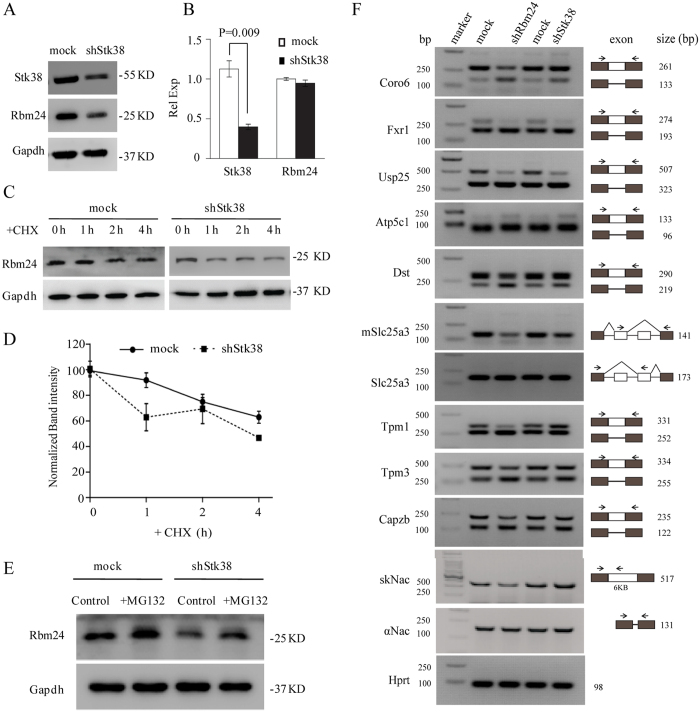
Stk38 affects Rbm24 protein stability and splicing activity. (**A**) HL-1 transfected with control vector or shStk38. Western blot was done using antibodies against Stk38, Rbm24, and Gapdh. Result shows that transfection with shStk38 was able to knockdown the protein level of Stk38, which in turn reduced the level of Rbm24 protein. (**B**) qRT-PCR analysis showed knockdown of Stk38 in HL-1 cells did not affect mRNA level of Rbm24, indicating that Stk38 did not regulate Rbm24 at the mRNA level. P-values were calculated using Student’s t-test with significance set at *p* < 0.05. Values where *p* < 0.05 are shown (n = 3). (**C**) HL-1 cells were transfected with shStk38 before the half-life of Rbm24 protein was analyzed by immunoblotting of total protein lysates harvested at the indicated times after inhibition of protein synthesis by CHX. (**D**) Densitometric analysis of Rbm24 level normalized with Gapdh protein level at each treatment time point with CHX as compared with the initial level of Rbm24. Result shows that reduction of Stk38 greatly reduced the level of Rbm24 overtime with CHX treatment, further justifying that Stk38 could be required for Rbm24 stability (n = 3). (**E**) HL-1 or HL-1-shStk38 cells were treated with MG132. Total cell lysates were subjected to western blot analysis with anti-Rbm24 and anti-Gapdh antibodies. Results show that Stk38 increases the stability of Rbm24 by preventing its degradation via ubiquitin-proteasome protein degradation pathway. (**F**) Splicing analysis for the indicated RNAs was performed by RT-PCR with HL-1-shRbm24 and HL-1-shStk38 cells. Primers and expected band sizes are indicated. Naca has two isoforms, skNAC and aNAC, generated by alternative splicing of exon 2. skNAC, which contains exon 2, is specifically expressed in muscle tissues, whereas aNAC is expressed ubiquitously. mSlc25a3 represents the muscle-specific isoform of Slc25a3. Hprt was used as an internal control. The images of (**A,C,E,F**) shown are cropped. The full-length blots or original images are shown in Figure S3.

To explore the potential role of Stk38 and Rbm24 interaction, we suppressed Stk38 expression by transfecting Stk38 shRNA to mouse HL-1 cells (HL-1-shStk38 cells), which exhibit the characteristics of differentiated cardiomyocytes with high expression of cardiac-specific proteins. As shown in Fig. 3, Stk38 shRNA silencing of endogenous Stk38 resulted in significant reduction in the level of Rbm24 protein (Fig. 3A), whereas Rbm24 mRNA level was not affected (Fig. 3B).

To verify the Stk38 shRNA-mediated reduction of Rbm24 protein level, we treated HL-1-shStk38 cells with cycloheximide (CHX) to block total cellular protein synthesis, and examined the stability of Rbm24 protein. As shown in Fig. 3C,D, knockdown of Stk38 dramatically reduced the half-life of Rbm24. In control cells (transfected with an empty vector alone), Rbm24 protein was highly stable during the period of CHX treatment. In contrast, Rbm24 level in HL-1-shStk38 cells was rapidly decreased within 1 h of CHX treatment, suggesting that Stk38 affects Rbm24 protein stability.

To investigate the mechanisms by which Stk38 influences Rbm24 protein stability, the HL-1 and HL-1-shStk38 cells were treated with the proteasome inhibitor MG132. As shown in Fig. 3E, shStk38 treatment decreased the Rbm24 protein level, while an MG132 treatment prevented the reduction of Rbm24 protein level. These results indicated that Stk38 increases Rbm24 protein stability probably by interfering with the ubiquitin-proteasome protein degradation pathway.

Previous studies showed that Rbm24 governs muscle-specific splicing events that are critically involved in cardiac development^9^,^10^. To examine whether the effect of Stk38 on Rbm24 protein stability would influence Rbm24′s splicing, we analyzed Rbm24-dependent splicing both in HL-1-shRbm24 and HL-1-shStk38 cells. Similar to the alternative splicing (AS) in previous studies^9^,^10^, Rbm24-regulated AS pattern was observed in HL-1-shRbm24 cells in all cases. As shown in Fig. 3F, Coro6, Fxr1, Usp25, Atp5c1, Dst, and mSlc25a3/Slc25a3 showed a reduction of Rbm24-dependent exons both in shRbm24 and shStk38 cells. Moderate AS changes of Tpm1, Tpm3, Capzb and skNAC/aNac were observed in shStk38 cells, presumably due to cell line specific effect or shStk38 treatment did not completely abrogate Rbm24 protein.

### Knockdown of Stk38 disrupts sarcomere assembly in cardiac cells

In our previous study, we identified a novel role of the tissue-specific Rbm24 involving in the regulation of sarcomere assembly and heart contractility^7^. Since our findings indicate that Stk38 could sustain Rbm24 protein stability, we sought to determine whether Stk38 could affect sarcomere assembly in cardiac cells.

**Figure 4:**
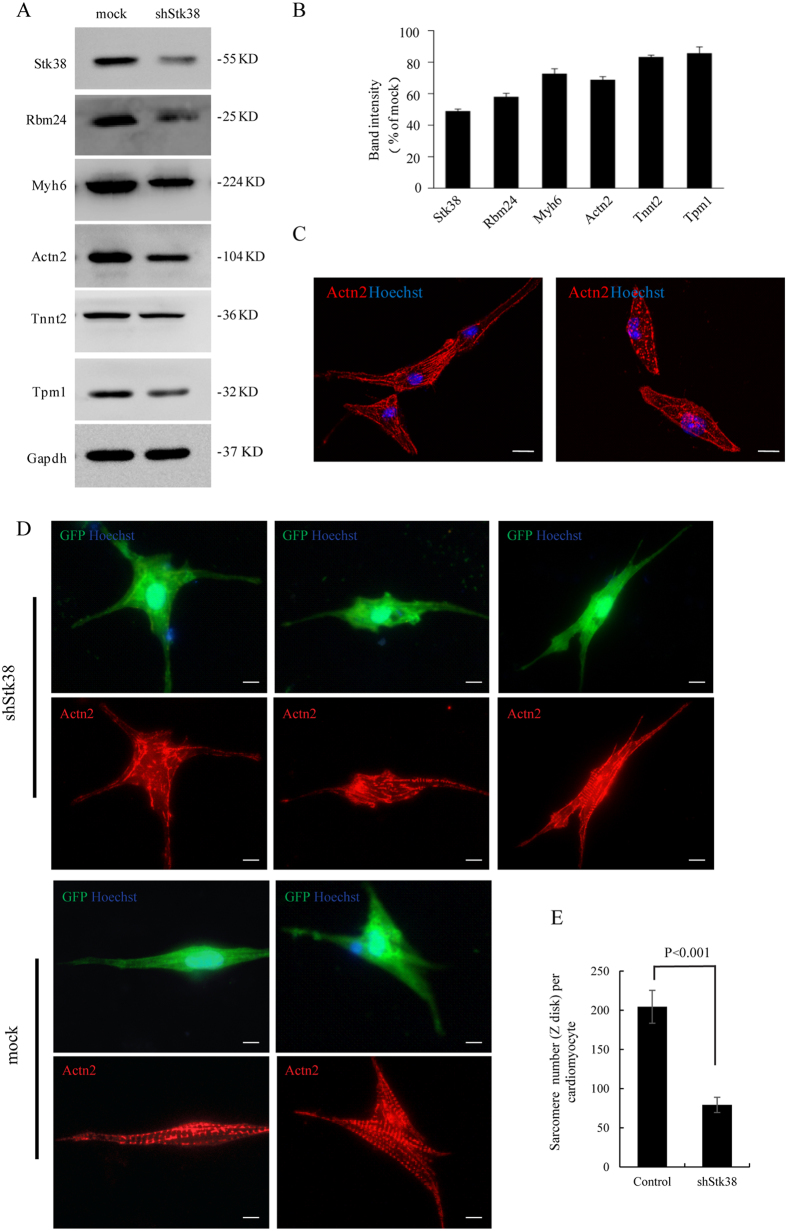
Stk38 is required for sarcomere assembly. (**A**) HL-1 cells were transfected with shStk38, then the expression of Stk38, Rbm24, Myh6, Actn2, Tnnt2 and Tpm1 were analyzed with western blot. Results show that Stk38 knockdown led to the decrease in expression of these cardiac proteins. (**B**) Densitometric analysis of Stk38, Rbm24, Myh6, Actn2, Tnnt2 and Tpm1protein levels normalized with Gapdh. (**C**) Immunofluorescence imaging of sarcomere (Actn2, red fluorescence) in HL-1 cells transfected with shStk38. (**D**) Immunofluorescence imaging of sarcomere (Actn2, red fluorescence) in primary cardiomyocytes transfected with shStk38. The direct GFP fluorescence is from shStk38 or empty lentivirus constructs co-expressing GFP protein. Result indicates the perturbation of Actn2 distribution and subsequent distortion to the thin filaments network from Stk38 knockdown cells. Bar = 12 μm. (**E**) Mean of sarcomere numbers per cardiomyocyte transfected with shStk38 or empty vector. Sarcomere numbers were counted >20 cardiomyocytes with a 600× objective. The image of A shown is cropped. The full-length blot or original image is shown in Figure S4A.

Firstly, we examined if the levels of cardiac proteins involved in sarcomere organization were changed after Stk38 was knocked down in HL-1 cells. As shown in Fig. 4A,B, Stk38 knockdown led to a reduction of cardiac proteins, including Myh6, Actn2, Tnnt2 and Tpm1. As these proteins are known to involve in sarcomeric organization, we next examined whether the sarcomere was affected in HL-1 cells. Actn2 contributes to Z-disk mechanics of sarcomere and the distribution of Actn2 was usually used to analyze sarcomeric substructure. As shown in Fig. 4C, the distribution of Actn2 is affected after Stk38 knockdown, indicating a disturbed Z-disk in HL-1-shStk38 cells.

Furthermore, we extended our analysis to primary cardiomyocytes isolated from neonatal mouse heart. A reduction in Stk38 as well as Rbm24 was observed 2 days after shStk38 lentivirus infection (Figure S4B). Immunofluorescence imaging of sarcomere (Actn2, red fluorescence) in primary cardiomyocytes transfected with shStk38 was analyzed. To ensure the transfection was effective, the GFP fluorescence from shStk38 or empty lentivirus constructs co-expressing GFP was used to identify transfected cardiomyocytes. We closely compared the sarcomere structure of control and Stk38 knockdown cells. Stk38 knockdown disrupts Actn2 distribution (Fig. 4D) and reduces the number of sarcomere per cell (Fig. 4E) in primary cardiomyocytes. These results suggested that Stk38 plays an important role in regulating the formation of a functional sarcomere.

We further explored whether a knockdown of Stk38 levels resulting in disturbed sarcomere structure could be rescued by over-expression of Rbm24. We firstly generated a stable shStk38 HL-1 cell line, and then introduced an exogenous Rbm24 into the shStk38 cells. The cardiac proteins involved in sarcomere organization were measured before and after Rbm24 over-expression. The Stk38 knockdown alone showed a reduction of Actn2, Tnnt2 and Tpm1 (Figure S5A), while the over-expression of Rbm24 rescued significantly the expression of these proteins. Moreover, over-expression of Rbm24 rescued the disturbed distribution of sarcomere results from deletion of Stk38 in primary cardiomyocytes was observed (Figure S5B). These results suggest Rbm24 could mediate the specific role of the Stk38 in regulating sarcomere assembly.

### Stk38 mediates sarcomere assembly through Rbm24 in a kinase-dependent way

Previous studies showed that treatment of cells with okadaic acid (OA), a potent inhibitor of protein phosphatase type 2A (PP2A), resulted in increased phosphorylation and activation of Stk38^23^,^24^,^25^,^26^. Importantly, purified PP2A is able to completely inactivate human Stk38 *in vitro*^27^, which indicates that OA has a direct effect on the phosphorylation and activation of Stk38. In contrast, the other compound 1,2-bis(o-aminophenoxy)ethane-N,N,N′,N′-tetraacetic acid tetra(acetoxymethyl)ester (BAPTA-AM) could effectively suppress the phosphorylation of Stk38 on both Ser-281 and Thr-444, thus impeding its activity^23^,^28^.

**Figure 5:**
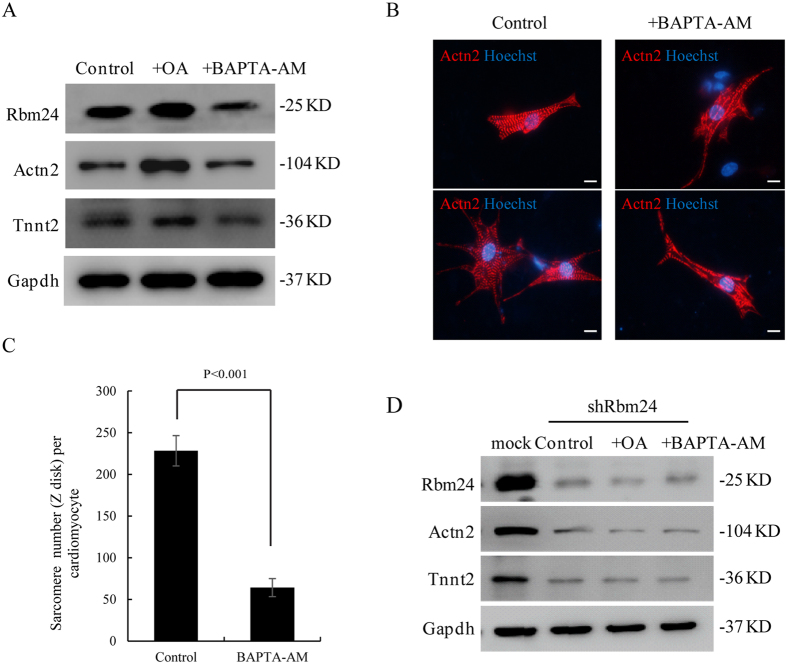
Rbm24 is required for Stk38-regulated sarcomere assembly. (**A**) HL-1 cells were pre-incubated with BAPTA-AM or OA before Rbm24 and sarcomeric proteins were analyzed with western blot. Results show that the alteration in the activity of Stk38 kinase affects the protein level of Rbm24, Actn2 and Tnnt2. (**B**) Immunofluorescence imaging of sarcomere (Actn2, red fluorescence) in primary cardiomyocytes treated with BAPTA-AM. Bar = 12 μm. (**C**) Mean of sarcomere numbers per cardiomyocyte treated with BAPTA-AM or not. Sarcomere numbers were counted >20 cardiomyocytes with a 600× objective. (**D**) HL-1 cells transfected with shRbm24 were incubated with BAPTA-AM or OA, then Rbm24 and sarcomeric proteins were analyzed with western blot. When Rbm24 was knocked down in HL-1 cells, the protein levels of Actn2 and Tnnt2 were not affected by alteration to the activity of Stk38, suggesting the role of Stk38-regulated sarcomere assembly is mediated by Rbm24. The images of A, D shown are cropped. The full-length blots or original images are shown in Figure S7.

Given that Stk38 kinase can be efficiently activated upon treatment of cells with OA or inhibited by BAPTA-AM, we used OA/BAPTA-AM to treat HL-1 cells to investigate whether Stk38 mediates sarcomere assembly in a kinase-dependent manner. We first examined the expression of cardiac proteins (Actn2 and Tnnt2) in the HL-1 cells treated by either OA or BAPTA-AM. As expected, OA/BAPTA-AM treatment strongly induced/reduced phosphorylation of Stk38 respectively (Figure S6). Importantly, OA treatment resulted in an up-regulation of Actn2 and Tnnt2 protein level, whereas BAPTA-AM treatment reduced the protein level of Actn2 and Tnnt2, as well as Rbm24 protein (Fig. 5A). Next, we examined the distribution of cardiac proteins by treating cells with BAPTA-AM. As shown in Fig. 5B,C, BAPTA-AM inactivation of Stk38 caused similar defects as observed in intracellular filaments with Stk38 knockdown, indicating that inactive Stk38 could perturb sarcomere formation of the heart.

Subsequently, we examined the cardiac proteins (Actn2 and Tnnt2) in HL-1-shRbm24 cells treated with OA or BAPTA-AM. As shown in Fig. 5D, regardless of either BAPTA-AM or OA treatment, Actn2 and Tnnt2 protein levels did not change in HL-1-shRbm24 cells, confirming the above result that Stk38 regulates sarcomere assembly through Rbm24.

### Stk38 sustains Rbm24 protein stability by phosphorylating Rbm24

**Figure 6:**
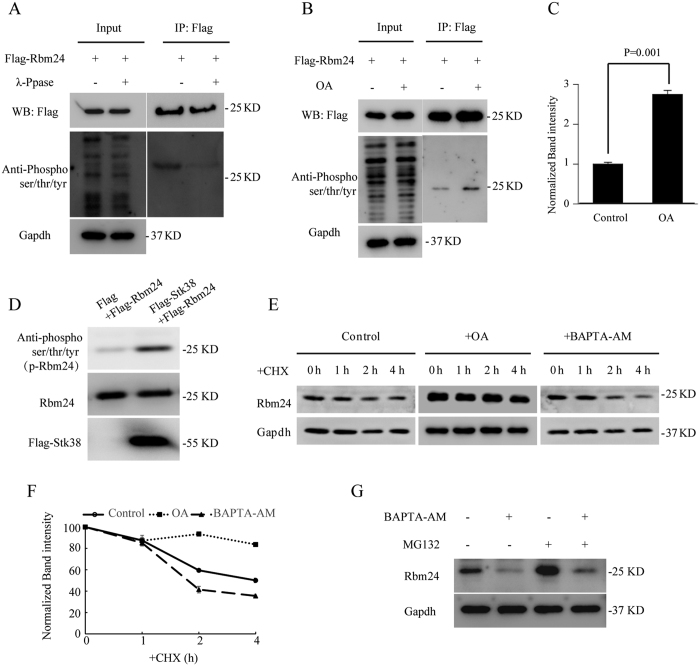
Stk38 affects Rbm24 protein stability by phosphorylating Rbm24. (**A**) Flag-Rbm24 was immunoprecipitated from λ-Ppase treated or not treated HEK293 cells that were transfected with Flag-Rbm24. (**B**) Activator of Stk38 (OA) increases the phosphorylation of Rbm24. Flag-Rbm24 was immunoprecipitated from OA-stimulated HEK293 cells that were transfected with Flag-Rbm24. Results show that the activation of Stk38 kinase activity led to the increase phosphorylation of Rbm24, demonstrating that Stk38 could phosphorylate Rbm24. (**C**) Densitometric analysis of phosphorylation of Rbm24 normalized with total Flag-Rbm24 protein level compared with Control. P-values were calculated using Student’s t-test with significance set at *p* < 0.05 (n = 3). (**D**) Stk38 phosphorylates Rbm24 *in vitro*. Flag-Stk38 (purified from HEK293 cells) was incubated with Flag-Rbm24 (purified from HEK293 cells) *in vitro*. The phosphorylated Rbm24 and total Rbm24 were detected by immunoblotting with the indicated antibodies. (**E**) HL-1 cells were treated with a combination of CHX and OA, or CHX and BAPTA-AM, before the half-life of Rbm24 protein was analyzed by immunoblotting of total protein lysates harvested at the indicated times. As shown in result, activating the kinase activity of Stk38 by OA increases the stability of Rbm24 in the presence of protein synthesis inhibitor CHX, whereas blocking the kinase activity of Stk38 by BAPTA-AM drastically reduces the level of Rbm24 in the presence of CHX. (**F**) Densitometric analysis of Rbm24 level normalized with Gapdh protein level for each time point compared to the initial level of Rbm24 protein. This result further highlights the role of Stk38 in stabilizing Rbm24 from degradation (n = 3). (**G**) HL-1 cells were treated with MG132 or a combination of MG132 and BAPTA-AM. Then total cell lysates were subjected to western blot analysis with anti-Rbm24 and anti-Gapdh antibody. The images of (**A,B,D,E,G**) shown are cropped. The full-length blots or original images are shown in Figure S9.

Previous studies showed that Stk38 can phosphorylate its downstream targets^14^,^24^,^29^, thus, we proceeded to investigate whether Rbm24 is phosphor-regulated by Stk38. We firstly applied in-gel phosphoprotein staining method to determine whether Rbm24 is a phosphoprotein. Flag-Rbm24 overexpressed in cells was immunoprecipitated with anti-Flag, followed by SDS-PAGE and sequentially stained with Pro-Q Diamond to visualize phosphoprotein, or with SYPRO Ruby to visualize all proteins^19^. Results showed that Pro-Q Diamond staining analysis could recognize Rbm24 (Figure S8), indicating that Rbm24 is a phosphorylated protein. In addition, we applied a phospho-ser/thr/tyr antibody to directly examine the phosphorylation of Rbm24 in cells treated with or without λ phosphatase (λ-Ppase). The corresponding western blot analysis (Fig. 6A) showed that treatment of the λ-Ppase resulted in dephosphorylation of Rbm24, further confirming that Rbm24 was phosphorylated.

Subsequently, we used OA to treat HEK293 cells overexpressing Flag-Rbm24 to examine whether Stk38 could influence the phosphorylation of Rbm24. As shown in Fig. 6B,C, the level of phosphorylated Rbm24 clearly increased after activation of Stk38 with OA. Next, we went on to perform an *in vitro* kinase assay to determine if Stk38 could directly phosphorylate Rbm24. Flag-Stk38 was pulled down and incubated with Flag-Rbm24 *in vitro*, which is followed by phospho-ser/thr/tyr antibody to detect whether Stk38 kinase could directly phosphorylate Rbm24. Our result showed that the level of phosphorylated Rbm24 was increased after incubation with Flag-Stk38 (Fig. 6D). This suggests that Stk38 could indeed directly phosphorylate Rbm24.

Considering that Stk38 regulates the stability of proteins through phosphorylation^30^,^31^, we next examined whether Stk38 sustains Rbm24 protein stability through phosphorylation. We tested the levels of Rbm24 protein over time in HL-1 cells treated with a combination of OA and CHX, or BAPTA-AM and CHX. As shown in Fig. 6E,F, Rbm24 protein level was decreased at 2 h after BAPTA-AM treatment, in contrast to the OA treatment, where Rbm24 became remarkably more stable as evident from its extended half-life beyond 4 h. These results suggest that inhibition of Rbm24 phosphorylation by BAPTA-AM treatment could contribute to the reduced half-life of Rbm24, whereas increased phosphorylation by OA treatment led to the increased half-life of Rbm24. Like the observation of MG132 treatment in Stk38 knockdown cells (Fig. 3E), MG132 treatment prevented the reduction of Rbm24 protein level with BAPTA-AM treatment (Fig. 6G), indicating that phosphorylation might be involved in the ubiquitin-proteasome protein degradation pathway of Rbm24.

## Discussion

We have previously characterized the functional role of Rbm24 in the regulation of cardiac gene expression, sarcomeric assembly and cardiac contractility in a zebrafish loss-of-function model^7^. A recent study in a mouse knockout model suggested that Rbm24 deficiency led to severe sarcomeric disarrangement in striated muscles of the mouse heart^10^, consistent with our observations in the zebrafish. These studies provided an insight into the tissue-specific role of Rbm24 in regulating the expression of cardiac structural proteins linked to the formation of the Z-disc and cardiomyopathy^7^. Understanding the exact mechanism by which it functions will depend on the identification of Rbm24′s regulators. In this paper, we found the *in vivo* binding of Rbm24 to Stk38 by co-immunoprecipitation studies and mass spectrometry analysis. We also found that the loss-of-function of Stk38 resulted in irregular sarcomere arrangement. Thus, our analysis defines a novel regulatory mechanism of Stk38-Rbm24 signaling in sarcomerogenesis and cardiac function.

Furthermore, we demonstrated that Stk38 regulates Rbm24 through sustaining the stability of Rbm24 protein level in a kinase activity-dependent manner. For the first time, our study identified Rbm24 as a phosphoprotein, and showed that its phosphorylation state could be modulated by Stk38. Such Stk38 phosphorylation could stabilize Rbm24 protein, and the degree of Rbm24 phosphorylation is important for its sarcomerogenesis function. Post-translational modification by phophorylation is a well characterized modification for RNA-binding proteins. It controls protein-protein interactions^32^, protein-RNA interactions^33^, splicing activities^34^,^35^, alters splicing factors intracellular localization^36^,^37^,^38^ and stability^39^. In this study, we have established Stk38 as an endogenous positive phosphor-regulator of Rbm24. It is of interest to identify the phosphosite(s) in Rbm24 protein. Bioinformatics analysis predicted 14 potential threonine/serine phosphorylation sites on the Rbm24 protein (http://kinasephos.mbc.nctu.edu.tw/predict.php), which could potentially be phosphorylated by Stk38. Future identification and validation of these phosphorylation sites of Rbm24 by the combination of bioinformatics approaches, mass spectrometry analysis, mutagenesis-based assay, as well as generation of phospho-specific antibodies could further aid in elucidating the post-translational modification regulatory mechanisms involved.

The assembly of sarcomeric proteins into the highly-organized structure of the sarcomere is an ordered and complex process. Sarcomeric dysfunction is both a cause and a consequence of contractile dysfunction, and is link to cardiomyopathy and heart failure^40^. Our data provide evidence that a deficient Stk38 could destabilize the Rbm24 protein, leading to abnormality in the distribution of sarcomeric proteins. This illustrates a sarcomere abnormality consistent with characteristics of cardiomyopathy developing in the Rbm24a-deficient myocardium^7^. Knockdown of Stk38 resulted in defective cardiac contractility as correlated with changes in the expression of sarcomere genes: Tnnt2, Tpm1, Actn2, Myh6 (Fig. 4A)^41^,^42^,^43^. These genes encode thin and thick filament, and the Z disk proteins of the sarcomeres, representing the cardiac contractility machinery^44^. Notably, the effects of Stk38 on sarcomere protein disappeared in shRbm24 cells, suggesting that Stk38 regulates the sarcomere through Rbm24. Phosphorylation of Rbm24 by Stk38 is crucial for the maintenance of cardiac sarcomeric gene expression in cardiac cells. Our data indicated that a deficient of Stk38/Rbm24 signaling leads to a significant defect in sarcomere assembly. Our study could better facilitate the understanding of the mechanisms of sarcomeric dysfunction related cardiac diseases.

Previous studies mainly focus the role of Stk38 on cell proliferation^20^, centrosome duplication^15^,^16^,^17^ and apoptosis^18^,^19^, closely correlating with Hippo tumor suppressor pathway^22^. Stk38 has been characterized as a regulator of c-myc and p21 protein stability through phosphorylation during cell cycle progression^14^,^29^. Only several reports have demonstrated changes in Stk38 expression with cytoskeleton based on genetic studies performed in fungus and flies, where its functions were involved in nuclear migration^45^ and normal actin dynamics^46^,^47^. It remains unclear whether Stk38 kinase can also regulate the sarcomere of the heart in mammals. Therefore, our study provides the first evidence that Stk38 is functional in sarcomere assembly through regulating Rbm24, hence revealing a previously unknown biological role of Stk38 in sarcomerogensis. Given the diverse roles Stk38 is involved in, it is possible that Stk38 may also control other physiological heart function through a different molecular mechanism in the heart, and it remains to be exploited whether additional pathways are involved in myocardium defects resulting from Stk38 deficiency.

**Figure 7:**
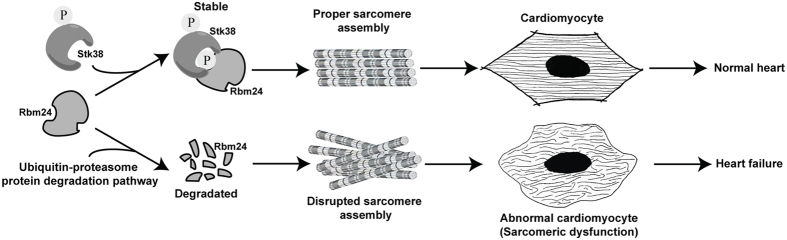
Working model for Stk38′s function in sarcomere assembly. Stk38 first binds to Rbm24 to induce its phosphorylation. Phosphorylated Rbm24 has greater stability and is less prone to protein degradation. The unaltered level of Rbm24 allows proper sarcomere assembly to occur.

Taken together, our study reveals a previously unknown biological role of Stk38 to be involved in sarcomere assembly and defines a novel regulatory mechanism of Stk38-Rbm24 signaling. We speculate that the binding of Stk38 to Rbm24 stimulates phosphorylation and stability of Rbm24, thus promoting proper sarcomere assembly in heart cells. A proposed working model for Stk38-Rbm24 signaling in sarcomere assembly in cardiomyocytes is shown in Fig. 7. The characterization of mechanism for the control of Rbm24 protein stability by Stk38 suggests a post-translational regulation of sarcomere assembly in cardiomyocytes. In conclusion, phosphorylation of Rbm24 by Stk38 is crucial for the maintenance of cardiac sarcomeric function in cardiac cells. These findings add to the understanding of the role of RBPs in sarcomere assembly and reveal a novel regulatory pathway in cardiogenesis that may deepen our understanding of the underlying causes of cardiomyopathy. Considering the importance of the phosphorylation state of protein in identifying the pathophysiological basis of sarcomeric dysfunctions, regulating the phosphorylation of Rbm24 could be a potential therapeutic strategy in heart diseases.

## Methods

An expanded material and methods section is given in the Supplementary material.

### Ethics statement

All experimental protocols were approved by the Institutional Animal Use and Care Committee (IACUC) of Xiamen University (Xiamen, Fujian, China; approval ID: SCXK2013-0006). The surgeries and procedures were performed in strict accordance with the Guidelines for the National Care and Use of Laboratory Animals by the National Animal Research Authority (China).

### Cells culture

H9C2 and HEK293 cells were cultured in DMEM (Invitrogen) supplemented with 10% fetal bovine serum (Geminin) and penicillin/streptomycin. AT-1 murine cardiomyocytes-derived HL-1 cells (gift from W. Claycomb, Louisiana State University) were maintained as previously described^48^. Briefly, cells were cultured on gelatin (0.02%, w/v)/fibronectin (10 μg/ml)-coated plates. The cells were maintained in Claycomb medium (Sigma Aldrich, Saint Louis, Missouri, USA) supplemented with 10% fetal bovine serum, 2 mM L-glutamine, 0.1 mM Norepinephrine, 100 U/ml penicillin, and 100 μg/ml streptomycin at 37 °C /5% CO_2_ in an incubator. The culture medium was changed every 24 h.

### Isolation and culture of primary cardiomyocytes

Male adult C57BL/6 mice were obtained from the Animal Research Center of Xiamen University. Neonatal mice around 24-hr-old were euthanized via CO_2_ inhalation and the hearts were excised for primary cardiomyocytes culture. Primary cultures of mouse cardiomyocytes were obtained by using a commercial isolation kit developed for neonatal ventricular myocytes (Worthington Biochemicals, Freehold, NJ). Overexpression, shRNA, or lentivirus constructs were transfected into primary cardiomyocytes (For details, see below).

### Constructs, transfection and lentiviral transduction

Silencing Stk38 shRNA (lentiviral vectors, pGLV-h1-GFP-Puro) was purchased from Genepharma. pXJ40-Myc-Rbm24 expression plasmid, pXJ40-Myc-C-terminal expression plasmid, pXJ40-Myc-RRM expression plasmid, pLV CS2.0N-Flag-Rbm24 lentiviral vector and pXJ40-Flag-Stk38 expression plasmid were obtained. For details, please refer to the supplementary material. Transient transfections were performed using Lipofectamine 2000 (Invitrogen) according to the manufacturer’s protocol. Silencing Stk38 shRNA or pLV CS2.0N-Flag-Rbm24 lentiviral vectors were co-transfected into HEK293 cells with pCMV-VSVG, pRSV-REV, and pMDL-g according to the manufacturer’s protocol. Primary cardiomocytes were infected with viral particles by centrifugation at 1200 rpm for 90 min, in the presence of 5 μg/ml polybrene (Sigma). Stable H9C2 cell lines expressing Flag-Rbm24 were selected with 2 μg/ml puromycin (Millipore).

### Immunoprecipitation and western blot

Cells were harvested for immunoprecipitation and western blot. For details, please refer to the supplementary material.

### In-gel digestion and mass spectrometric protein identification

The protein band was excised from the gel and subjected to mass spectrometry analysis. Protein identification was performed with the ProteinPilot 4.5 software Revision 1656 (SCIEX). Proteins identified with unused score ≥1.3 (confidence interval ≥95%) were deemed as positive identification. For details, please refer to the supplementary material.

### Immunofluorescence

Cells were fixed and proceeded for immunofluorescence detection. Refer to the Supplementary material for details.

### Sarcomere Assays

Sarcomere assay was performed as previously described^49^. We stained sarcomere Z disc with fluorescently labeled Actn2 and calculated sarcomere number in primary cardiomyocytes. Fluorescence microscopy was carried out with Olympus fluorescence microscope equipped with a 600× objective. The Actn2 fluorescence signals, represented sarcomere (Z disc) numbers, were counted >20 cardiomyocytes in blind assays. In total, 20–30 mice were used for each condition.

### Alternative splicing assay

Alternative splicing assay was performed as previously described^9^. Specific primers flanking predicted sites of alternative splicing were used for RT-PCR amplifications. PCR products were separated by high-resolution agarose gel electrophoresis. Primers were listed in Supplemental Table S1.

### Real-time PCR

Total RNA from the cells was extracted by using Trizol and further purified with RNeasy kit (Qiagen, Germany). RNA was then used for reverse transcription by following the protocol of the SuperScript III First Strand Kit (Invitrogen, USA). Real-time PCR was used to quantify Rbm24 and Stk38. The real-time PCR was performed with the ABI 7500 machine. Primers were listed in Supplemental Table S2.

### In-gel phosphoprotein staining

The proteins were separated using standard polyacrylamide electrophoresis techniques. Then, the gel was stained for phosphoprotein examination. For details, please refer to the supplementary material.

### *In vitro* kinase assay

For the *in vitro* kinase assay, HEK293 cells were transfected with plasmids expressing Flag-Rbm24 or Flag-Stk38. 48 hours after transfection, Flag-Rbm24 and Flag-Stk38 was purified. Then, Flag-Rbm24, Flag-Stk38 and ATP were kept in the kinase assay buffer for 30 min at 30 °C followed by immunoblotting with the anti-phospho-ser/thr/tyr antibody.

### Statistical analysis

Results are presented as mean ± SEM unless otherwise specified. Comparison between groups was performed by Student’s t-test. All statistical procedures were performed using SPSS software. Differences were considered significant at a value of *p* < 0.05.

## Additional Information

**How to cite this article:** Liu, J. *et al*. Stk38 Modulates Rbm24 Protein Stability to Regulate Sarcomere Assembly in Cardiomyocytes. *Sci. Rep.* **7**, 44870; doi: 10.1038/srep44870 (2017).

**Publisher's note:** Springer Nature remains neutral with regard to jurisdictional claims in published maps and institutional affiliations.

## Supplementary Material

Supplementary Data
