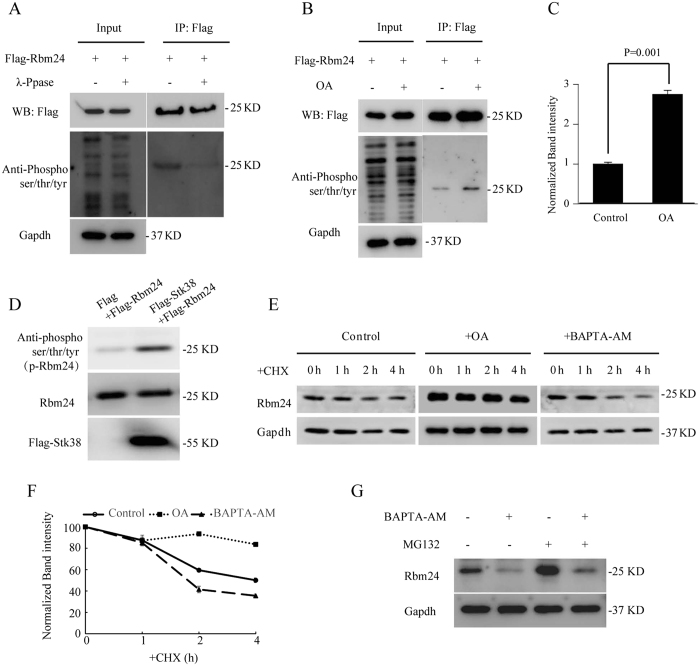# Corrigendum: Stk38 Modulates Rbm24 Protein Stability to Regulate Sarcomere Assembly in Cardiomyocytes

**DOI:** 10.1038/srep46854

**Published:** 2017-06-30

**Authors:** Jing Liu, Xu Kong, Yew Mun Lee, Meng Kai Zhang, Li Yan Guo, Yu Lin, Teck Kwang Lim, Qingsong Lin, Xiu Qin Xu

Scientific Reports 7: Article number: 44870; 10.1038/srep44870 published online: 03 21 2017; updated: 06 30 2017.

The original version of this Article contained errors.

The original version contained errors in the spelling of the authors Yew Mun Lee, Meng Kai Zhang and Li Yan Guo, which were incorrectly given as Lee Yew Mun, Zhang Meng Kai and Guo Li Yan.

In addition, the labels for Figure 4C were obstructed by Figure 4B. The correct Figure 4 appears below as Figure 1.

Furthermore, in Figure 6, the protein band label 25KD for ‘Anti-Phospho ser/thr/tyr’ was placed incorrectly. The correct Figure 6 appears below as Figure 2.

These errors have now been corrected in the HTML and PDF versions of this Article.

## Figures and Tables

**Figure 1:**
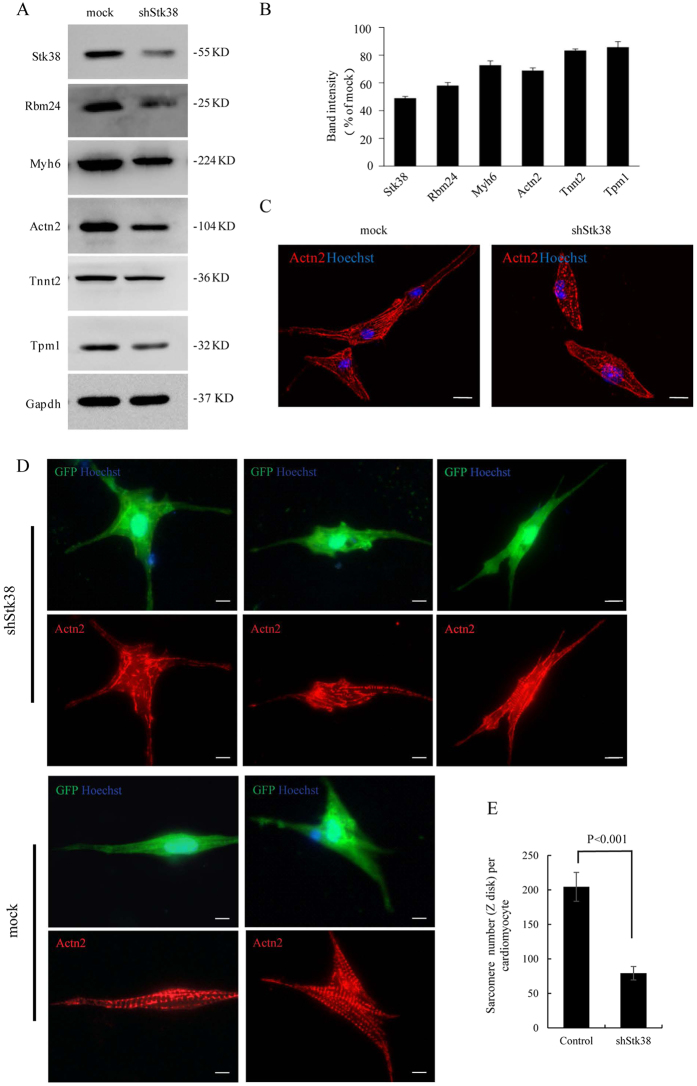


**Figure 2:**