# Supplementary material for: Stk38 Modulates Rbm24 Protein Stability to Regulate Sarcomere Assembly in Cardiomyocytes
Source: Sci Rep. 2017 Mar 21;7:44870. doi: 10.1038/srep44870 (PMC5359592; doi:10.1038/srep44870)
Supplement: Supplementary Data [file srep44870-s1.pdf]

Stk38 Modulates Rbm24 Protein Stability to Regulate Sarcomere Assembly in Cardiomyocytes

Jing Liu[1#], Xu Kong[1#], Yew Mun Lee [2], Meng Kai Zhang[1], Li Yan Guo[1], Yu Lin[1], Teck Kwang Lim [2], Qingsong Lin[2*], Xiu Qin Xu[1*]

[1] The Institute of Stem Cell and Regenerative Medicine, Medical College, Xiamen University, 361100, P.R. China.

[2] Department of Biological Sciences, National University of Singapore, 117543, Singapore.

* Correspondence to Xiu Qin Xu, Ph.D, The Institute of Stem Cell and Regenerative Medicine, Medical College, Xiamen University, Chengzhi Building, Xiang'an Campus, Xiamen, Fujian Province, 361100, P.R. China. Email: xuxq@xmu.edu.cn; Tel: +86-5922185276; Fax: +86-5922182736; Qingsong Lin, Ph.D, Department of Biological Sciences, National University of Singapore, 117543, Singapore. Email: dbslinqs@nus.edu.sg; Tel: +65-65167769; Fax: +65-67792486.

[#] These authors contributed equally to this work.

Supplemental data

1. Expanded Material and Methods

1.1 Constructs

Human Rbm24 coding sequence was obtained by RT-PCR using the primers: forward 5'-CTCGAGATGCACACGACCCAGAAGGAC-3' and reverse 5'-GGTACCCTATTGCATTCGGTCTGTCTGC-3', containing restriction sites for XhoI and KpnI, or 5'-CATATGATGCACACGACCCAGAAGGAC-3' and reverse 5'-ACTAGTCTATTGCATTCGGTCTGTCTGC-3', containing restriction sites for NdeI and SpeI, respectively. The PCR product was cloned in XhoI/KpnI sites of pXJ40-Myc plasmid to obtain the pXJ40-Myc-Rbm24 expression plasmid and in NdeI/SpeI sites of pLV CS2.0N-Flag plasmid to obtain the Rbm24 expression lentiviral vector. Myc-RRM and Myc-C-terminal were obtained by PCR from pXJ40-Myc-Rbm24 and cloned in XhoI/KpnI sites of pXJ40-Myc vector. Stk38 coding sequence was obtained by RT-PCR from H9C2 cell total RNA using the primers: forward 5'-GGATCCATGGCAATGACAGGATCTACACC and reverse 5'-CTCGAGCTACTTGGCCGCCTTCATGTAG-3' and subcloned in BamHI/XhoI sites of pXJ40-Flag plasmid to obtain the pXJ40-Flag-Stk38 expression plasmid.

1.2 Immunoprecipitation and western blot

Cells were harvested from plates by scraping after 2 washes with cold PBS. Then cells were centrifuged at 1,000 rpm for 15 min at 4°C and lysed by lysis buffer containing 50 mM Tris (pH 8.0), 150 mM NaCl, 10% glycerol, 0.2% NP-40, and protease inhibitor cocktail (Sigma). After the cells were centrifuged at 14,000 rpm for 15 min at 4°C, the supernatant was transferred to a clean Falcon tube and was added to Anti-Flag M2 (Sigma M8823), anti-Rbm24 or anti-rabbit IgG Magnetic Beads overnight. The beads conjugated with protein was then washed with 3×Flag peptide (Sigma F4799) in TBS buffer following washing 4 times with washing buffer containing 150 mM NaCl, 50 mM Tris (PH 8.0), 5% glycerol and 0.1% NP40. Then, the proteins were dissolved in loading buffer. 1/20 of each sample was loaded on and processed for western blot or for silver staining, the following antibodies were used: Anti-Flag M2 monoclonal antibody (Sigma, USA, F1804, 1: 1000), Anti-c-Myc Mouse monoclonal antibody (Transgen, China, HT101, 1: 4000), Anti-Rbm24 antibody (Abcam, England, ab94567, 1: 1000), anti-Stk38 monoclonal antibody (Abnova, Taiwan, H00011329-M02, 1: 500), anti-Actn2 (Sigma, USA, A7811, 1: 2500), anti-Tnnt2 (Sigma, USA, T6277, 1: 200), anti-Tpm1 (Sigma, USA, T2780, 1: 1000), anti-Myh6 (R&D Systems, MAB4470, 1: 1000), Anti-phospho-ser/thr/tyr antibody (Abcam, England, ab15556, 1;1000) and anti-Gapdh (Transgen, China, HC301, 1: 5000).

For phosphorylation examination, the protocol is the same as above, except the lysis buffer

and washing buffer was supplemented with 50 mM NaF and 1 mM Na_3VO_4.

1.3 In-gel digestion and mass spectrometric protein identification

The protein band was excised from the gel, diced into small pieces of about 1 mm^3 each and then incubated with 50 mM NH_4HCO_3/50% acetonitrile (ACN) for three times, 5 min each. The gel pieces were then dehydrated with ACN followed by vacuum-dry. The sample was then reduced with 10 mM DTT for 1 h at 57ºC and alkylated with 55 mM iodoacetamide (IAA) at room temperature for 1 h in the dark. The gel pieces were then alternatively washed with 100 mM ammonium bicarbonate and dehydrated with ACN twice. Finally, the sample was vacuum-dried and rehydrated with digestion solution containing 12.5 ng/µl sequencing grade modified trypsin (Promega) and 50 mM NH_4HCO_3, and incubated at 37 ºC for 16 h. Tryptic digest was extracted twice using 50% ACN and 0.1% TFA. The combined extract was desalted with Sep-Pak tC 18 uElution Plate (Waters, Miltford, MA, USA) and reconstituted with 20 µl of diluent (2% ACN with 0.05% Formic acid) and subjected to liquid chromatography-mass spectrometry (LC-MS) analysis.

The LC system used is an Eksigentnano LC Ultra coupled with ChiP LC-nanoflex (Eksigent, Dublin, CA, USA), in Trap-Elute configuaration. Both the trap column (200 µm × 0.5 mm) and the analytical column (75 µm × 150 mm) were made of ChromXP C18-CL, 3 µm (Eksigent, Germany). Five microliters of the sample was injected and the peptides were separated by a gradient formed by 2% ACN, 0.1% FA (mobile phase A) and 98% ACN, 0.1% FA (mobilephase B): 5 to 7% B in 0.1 min, 7 to 30% B in 10 min, 30 to 60% B in 4 min, 60 to 90% B in 1 min, maintaining at 90% B for 5 min, 90 to 5% B in 1 min and maintaining at 5% B for10 min, with a flow rate of 300 nL/min.

The MS analysis was performed with a TripleTOF 5600 system (SCIEX, Foster City, CA, USA) in Information Dependent Mode. MS spectra were acquired across the mass range of 400–1800 m/z in high resolution mode (>30000) using 250 ms accumulation time per spectrum. Tandem mass spectra were recorded in high sensitivity mode (resolution >15000) with rolling collision energy. In each duty cycle, a maximum of 20 precursors were selected for fragmentation, with 100 ms minimum accumulation time for each precursor and dynamic exclusion for 15 s.

Protein identification was performed with the ProteinPilot 4.5 software Revision 1656 (SCIEX) using the Paragon database search algorithm (4.5.0.0.1654), against the International Protein Index (IPI) Rat V 3.87 database (total 39923 entries). The parameter settings were: Sample Type - Identification; Cys Alkylation - Iodoacetamide; Digestion -trypsin; Special

Factors -None; Species-Rattus Norvegicus; ID Focus-Biological Modifications; Search Effort-Thorough; Detected Protein Threshold-0.05 (10.0%). Peptides identified with unused score ≥ 1.3 (confidence interval ⩾ 95%) were deemed as positive identification.

1.4 Immunofluorescence

Cells were fixed in 4% paraformaldehyde for 15 min, then washed three times with PBS and incubated with anti-Actn2 (Sigma, USA, A7811, 1: 800), anti-Stk38 (Abnova, Taiwan, H00011329-M02, 1: 500), anti-Tnnt2 (Sigma, USA, T6277, 1: 500) or anti-Rbm24 antibody (Abcam, English, ab94567, 1: 500) for overnight at 4°C. After incubation with primary antibodies, cells or sections were rinsed with PBS three times, for 5 min each. Then Alexa Fluor® 488 conjugated goat anti-rabbit IgG (H+L), or Alexa Fluor® 555 conjugated goat anti-mouse IgG (H+L) secondary antibody (Invitrogen, USA) was diluted 1: 500 and applied to cells respectively for 1 h at room temperature in the dark. Cells or sections were subsequently washed in PBS three times (5 min), and cell nuclei were counterstained with Hoechst 33258 (Sigma, USA, 1: 1000). Fluorescence was examined with microscope.

1.5 In-gel phosphoprotein staining

Proteins were separated using standard polyacrylamide electrophoresis techniques. The Tris-glycine gel was stained with Pro-Q solution or SYPRO Ruby solution (Invitrogen) according to the manufacturer's instructions. In briefly, the gel was incubated in fix solution (50% methanol and 10% acetic acid) for 30 min followed by a second overnight incubation. The next day, the gel was washed 3 times in ultrapure water, stained in Pro-Q dye for 70 min in the dark, and destained in a solution containing 20% acetonitrile and 50 mM sodium acetate (pH 4.0). After twice wash in ultrapure water, the gel was imaged on ChemiDoc MP (Bio-rad) using excitation at 580 nm. Subsequently, the Tris-glycine gel was stained in SYPRO Ruby protein gel stain solution.

2. Supplementary Tables

2.1 Table 1 RT-PCR primers are listed as follows:

Name	Forward Primer	Reverse Primer
Tpm1	GGGCTGAGTTGCAGAGAGATC	GTTTATTTTACACTGGGCGAATTG
Tpm3	CTGAAAGATCGGTAGCCAAGCT	GGAGGGAAAGCAGCCTTCAG
skNac	AAGCTGTGTCTGCATCTGTG	CTAACTGTGCTTGCTGAGAC
aNac	TACAGAGCAGGAGTTGCCAC	CTAACTGTGCTTGCTGAGAC
Hprt	TGATGAACCAGGTTATGACC	GTTGAGAGATCATCTCCACC
Itga6	GGTTCGAGTGACGGTGTTTCC	CATTCCACTTGGTGATCCACTG
Atp5c1	AGGCTGTCATCACAAAGGAGTTG	CTGTCTTTGTAACTCAGTGGACCAA
Capzb	ACAAAGGACATCGTCAACGG	AGAGGTCTTCAACACTGCTG
Coro6	TCATCATCTGGAATGTGGGC	GTACCGAATGCTACTTCAC
mSlc25a3	ACACCTGCAGCTGGTGCACG	CAGAGGAGAAAGAATCTGCC
Slc25a3	ACACCTGCAGCTGGTGCACG	GTGTCAAGCCCACAACTTAAG
Usp25	AGCAGCCATCAAGAAGTGAC	GTTTGGCTTGGGCAACTTTC
Dst	GTGATGGTTCGTGTTGGAGGC	CTCGACGCGGAAGTGGATCT
Fxr1	TCTCACCACAGTACTAACCG	TAGCCAAAGTTTCCCTTGGG

2.2 Table 2 Real-time PCR primers are listed as follows:

Name	Forward Primer	Reverse Primer
Gapdh	5'-CAATGTGTCCGTCGTGGATC-3'	5'-CCTGCTTCACCACCTTCTTG-3'
Rbm24	5'-ACTTGGGAGCAAAACCAAGA-3'	5'-GAAGCTGTTGAACGCCAAA-3'
Stk38	5'CAGAGATATCAAGCCAGACAACC3'	5'-AAAGGCCAAAGTCGGAAAGT-3'

3. Supplementary Figure and Figure legends

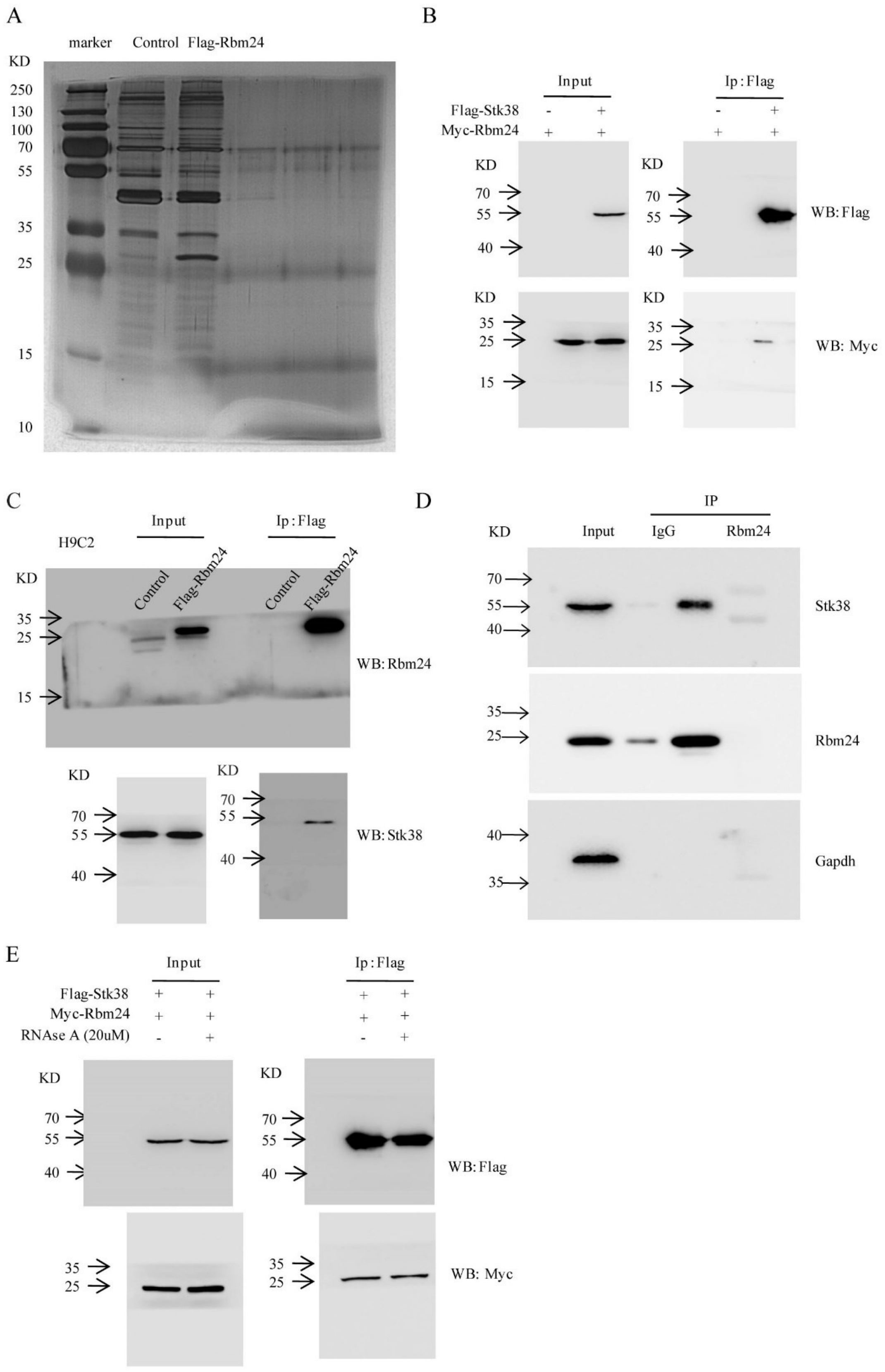

Figure S1. (A-E) The full-length gel/blots or original images for Figure 1A, C, D, E, F.

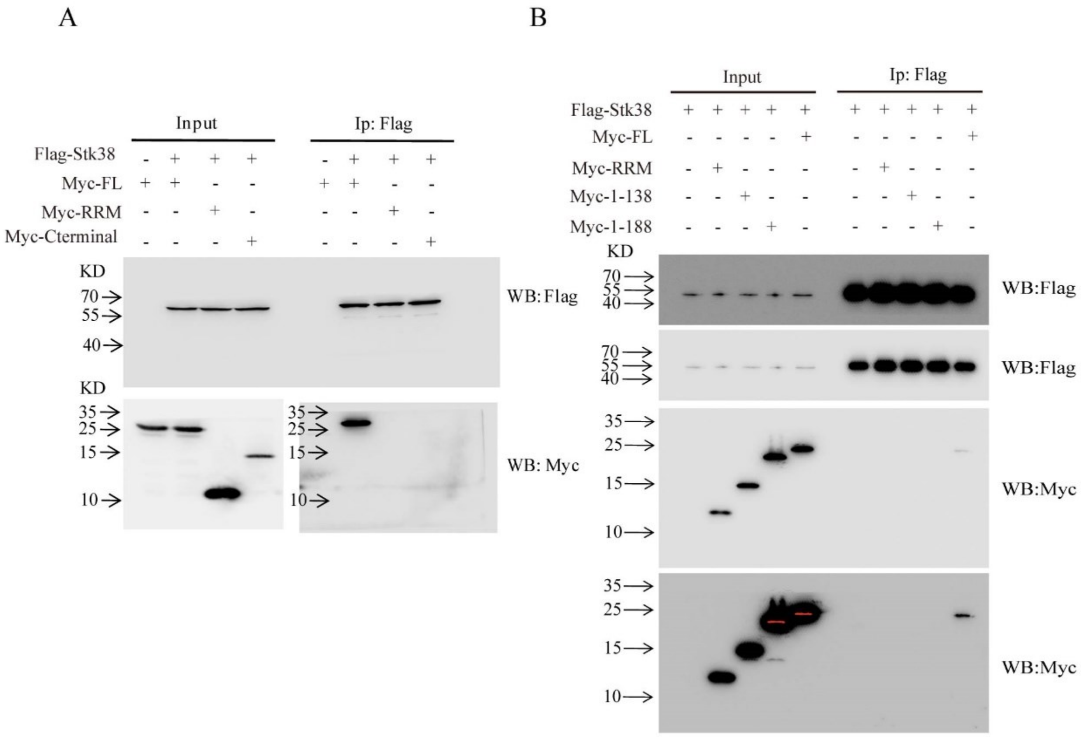

Figure S2. (A-B) The full-length blots or original images for Figure 2B and 2C.

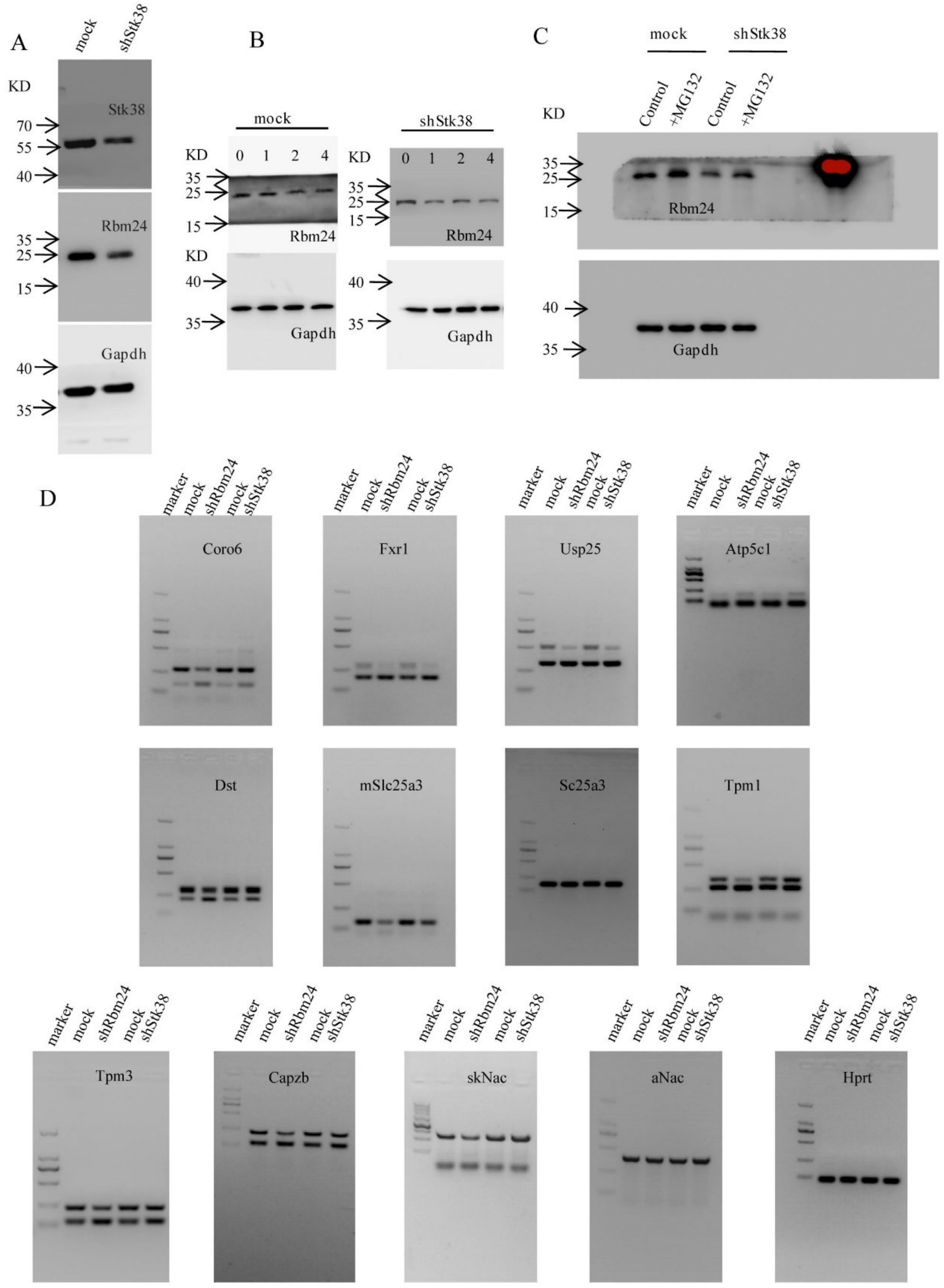

Figure S3. (A-D) The full-length blots or original images for Figure 3A, C, E and F.

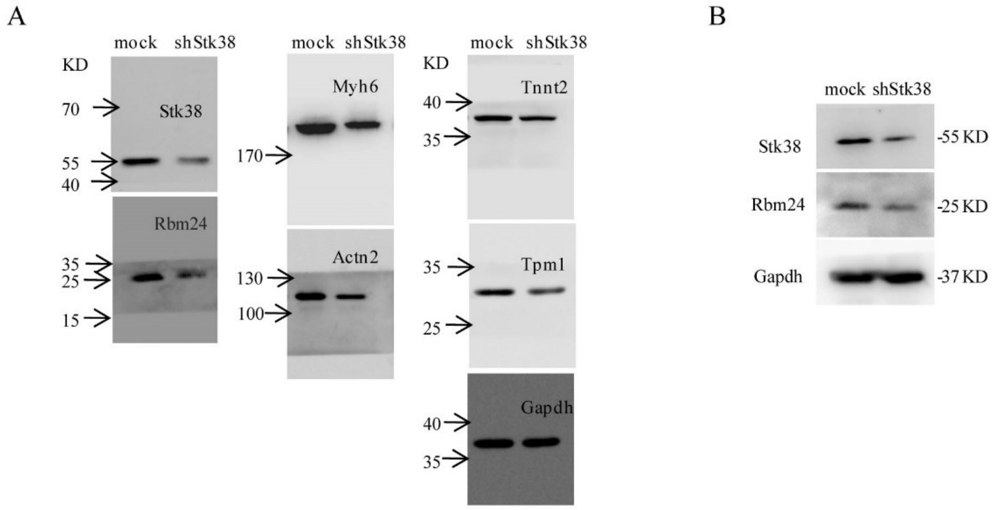

Figure S4. A. The full-length blots or original images for Figure 4A. B**.** Primary cardiomyocytes were transfected with shStk38, then the expression of Stk38 and Rbm24 were analyzed with western blot.

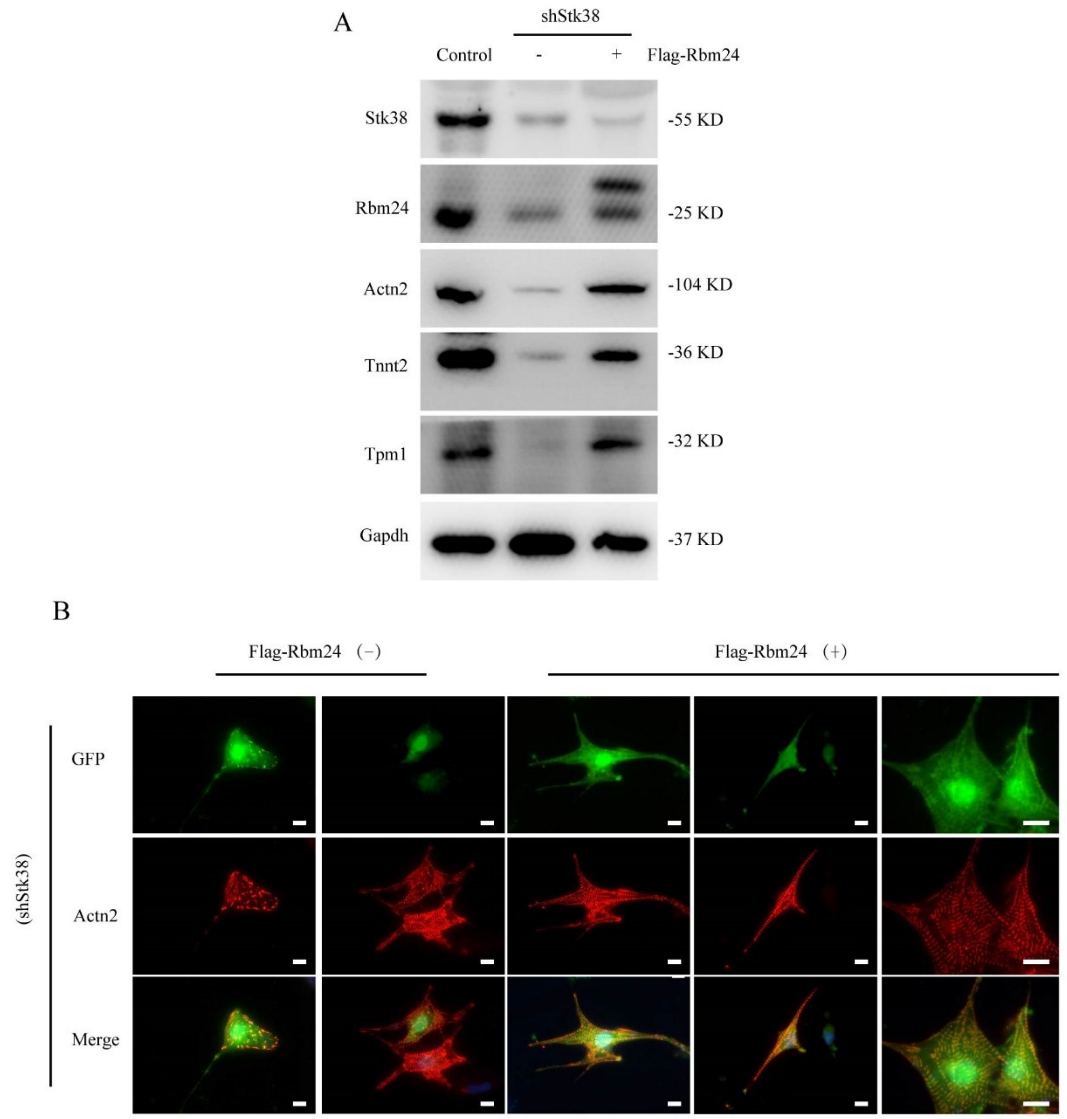

Figure S5. Overexpression of Rbm24 restores the phenotypic change mediated by shStk38. (A) HL-1 cells were co-transfected with Stk38 shRNA vector or control vector and vector expressing Rbm24 respectively, then the expression of Stk38, Rbm24, Actn2, Tnnt2 and Tpm1 were analyzed with western blot. (B) Immunofluorescence imaging of sarcomeric proteins Actn2 in primary cardiomyocytes co-transfected with Stk38 shRNA vector or control vector and vector expressing Rbm24 respectively. Bar = 12 μm.

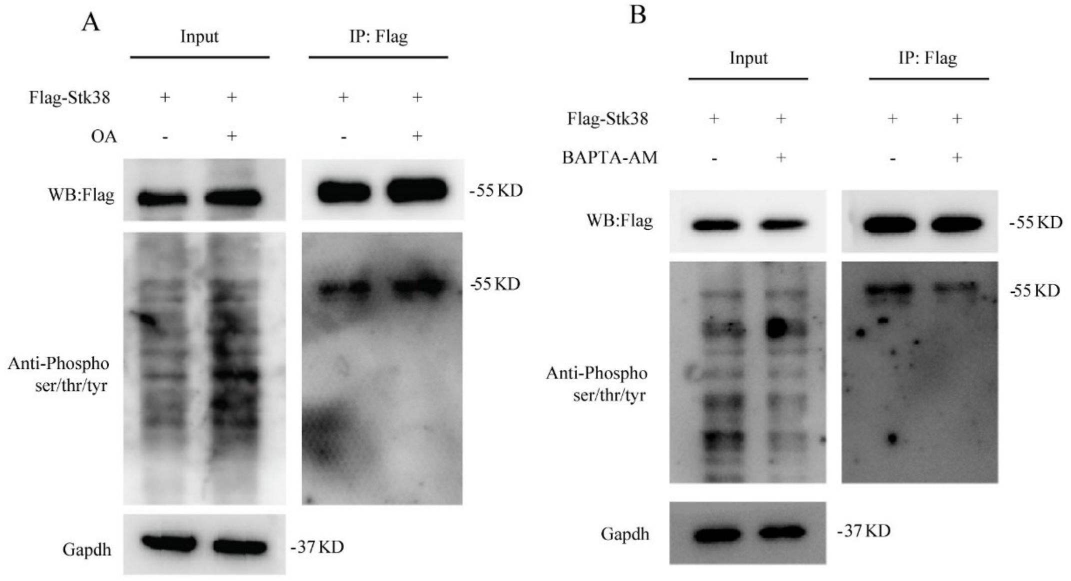

Figure S6. OA/BAPTA-AM treatment strongly induces/reduces phosphorylation of Stk38. Flag-Stk38 was immunoprecipitated from HEK293 cells that were transfected with Flag-Stk38. Results show that OA treatment led to an increase phosphorylation of Stk38, and BAPTA-AM treatment led to a decreased phosphorylation of Stk38.

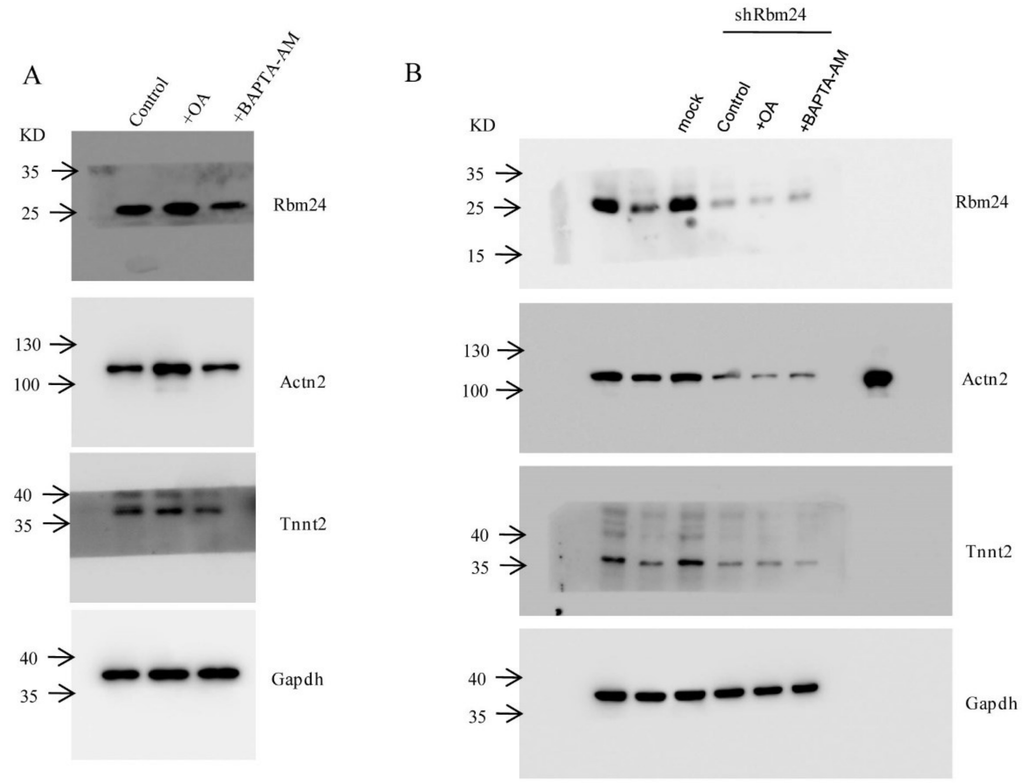

Figure S7. The full-length blots or original images for Figure 5A, D.

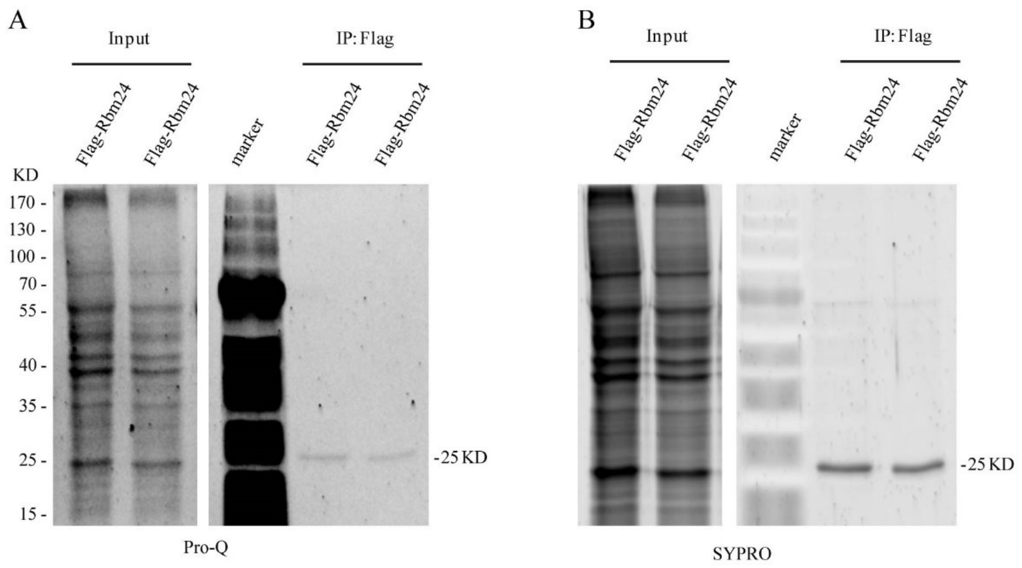

Figure S8. Rbm24 is a phosphorylation protein. Lysates from H9C2 cells overexpressing Flag-Rbm24 were subjected to immunoprecipitation with anti-Flag antibody, separated with SDS-PAGE, and lastly stained with Pro-Q to visualize phosphoprotein (left panel), or with SYPRO Ruby to visualize all proteins (right panel).

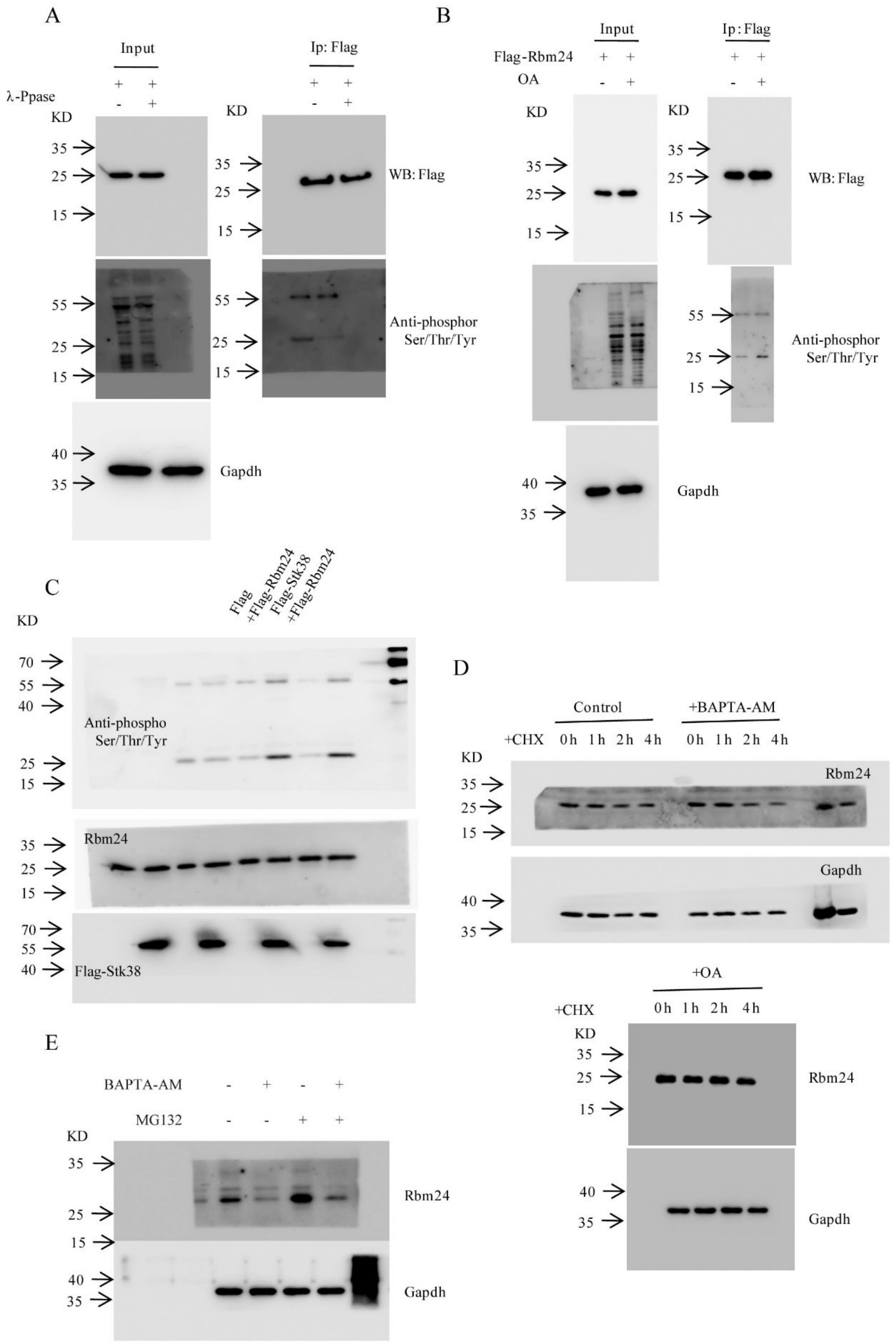

Figure S9. (A-E) The full-length blots or original images for Figure 6A, B, D, E and G.
